# FTY720 enhances TRAIL-mediated apoptosis by up-regulating DR5 and down-regulating Mcl-1 in cancer cells

**DOI:** 10.18632/oncotarget.3426

**Published:** 2015-03-23

**Authors:** Seon Min Woo, Bo Ram Seo, Kyoung-jin Min, Taeg Kyu Kwon

**Affiliations:** ^1^ Department of Immunology, School of Medicine, Keimyung University, 2800 Dalgubeoldaero, Dalseo-Gu, Daegu 704–701, South Korea

**Keywords:** FTY720, TRAIL, DR5, Mcl-1, apoptosis

## Abstract

FTY720, Fingolimod, is a functional antagonist to the sphingosine-1-phoaphate (S1P) receptor and an inhibitor of sphingosine kinase 1. Here, we showed that a combination of FTY720 and TRAIL induced apoptosis in human renal, breast, and colon carcinoma cells. Most importantly, this combination had no effect on normal cells. Furthermore, the combined treatment with FTY720 and TRAIL reduced tumor growth in xenograft models. FTY720 up-regulated death receptor (DR)5 at post-translational level. Knockdown of DR5 markedly blocked apoptosis induced by the combined treatment. FTY720 also inhibited Mcl-1 expression at the post-translational level. Over-expression of Mcl-1 blocked apoptosis induced by FTY720 and TRAIL. Interestingly, phospho-FTY720 and inhibitors of sphingosine kinase failed to enhance TRAIL-induced apoptosis. Thus, FTY720 enables TRAIL-induced apoptosis through up-regulation of DR5 and down-regulation of Mcl-1 in human cancer cells.

## INTRODUCTION

Sphingosine-1-phosphate (S1P) increases cancer cell proliferation [1, 2] and tumorigenesis [3, 4] and reduces cancer cell death [5]. FTY720 is a synthetic sphingosine analogue and is phosphorylated by sphingosine kinase 2 [6]. Phospho-FTY720 binds sphingosine-1-phosphate (S1P) receptors and induces the internalization of S1P receptors. Therefore, FTY720 acts as a functional antagonist [7]. The immunosuppressant effects of FTY720 are well known. Among the S1P receptors, S1P_1_ plays a crucial role in modulating lymphocyte migration and trafficking. Phospho-FTY720 binds S1P_1_ and then inhibits T lymphocyte egress from secondary lymphoid organs and migration into the transplanted graft, thereby suppressing inflammation [8]. In addition, novel functions of FTY720 have been reported. FTY720 induces cell death in multiple cancer cells, including cells from leukemia [9, 10], prostate [11], ovarian [12], and pancreatic [13] lines. Furthermore, FTY720 also sensitizes prostate cancer cells to radiotherapy [14], melanoma cells to cisplatin [15], and colon cancer cells to doxorubicin and etoposide [16]. Multiple FTY720-mediated apoptotic signaling pathways are independent of S1P signaling. The induction of protein phosphatase 2A [17], phospholipase C [18], and protein kinase C (PKC) activity was proposed to be involved in anti-cancer effects by FTY720.

Tumor necrosis factor-related apoptosis-inducing ligand (TRAIL) is a known inducer of apoptosis in cancer cells but not normal cells [19]. When TRAIL induces cell death, it binds to death receptor (DR) 4 and DR5, which have increased expression levels relative to normal cells [20]. In contrast, normal cells highly express decoy receptor (DcR) 1 and DcR2, such that this death-signaling pathway is unable to activate intracellular apoptotic signaling [21–24]. However, the down-regulation of DR expression, the up-regulation of anti-apoptotic proteins expression (c-FLIP(L), Bcl-2 and Bcl-xL) and the up-regulation of inhibitor of apoptosis proteins (IAPs) lead to resistance to TRAIL-mediated apoptosis in many cancer cells [25–29]. There are many studies that demonstrated related mechanisms of synergy between TRAIL and numerous agents [30–39]. Therefore, combination treatment with the TRAIL sensitizer could overcome TRAIL resistance.

In this study, we investigated whether FTY720 sensitized human renal carcinoma Caki cells to TRAIL-mediated apoptosis. We found that FTY720 enhanced TRAIL-mediated apoptosis in Caki cells through the up-regulation of DR5 and down-regulation of Mcl-1 expression. Collectively, our results suggest that combination treatment with FTY720 and TRAIL might be an effective therapeutic strategy for cancer treatment.

## RESULTS

### Combined treatment with FTY720 and TRAIL induces apoptosis

**Figure 1:**
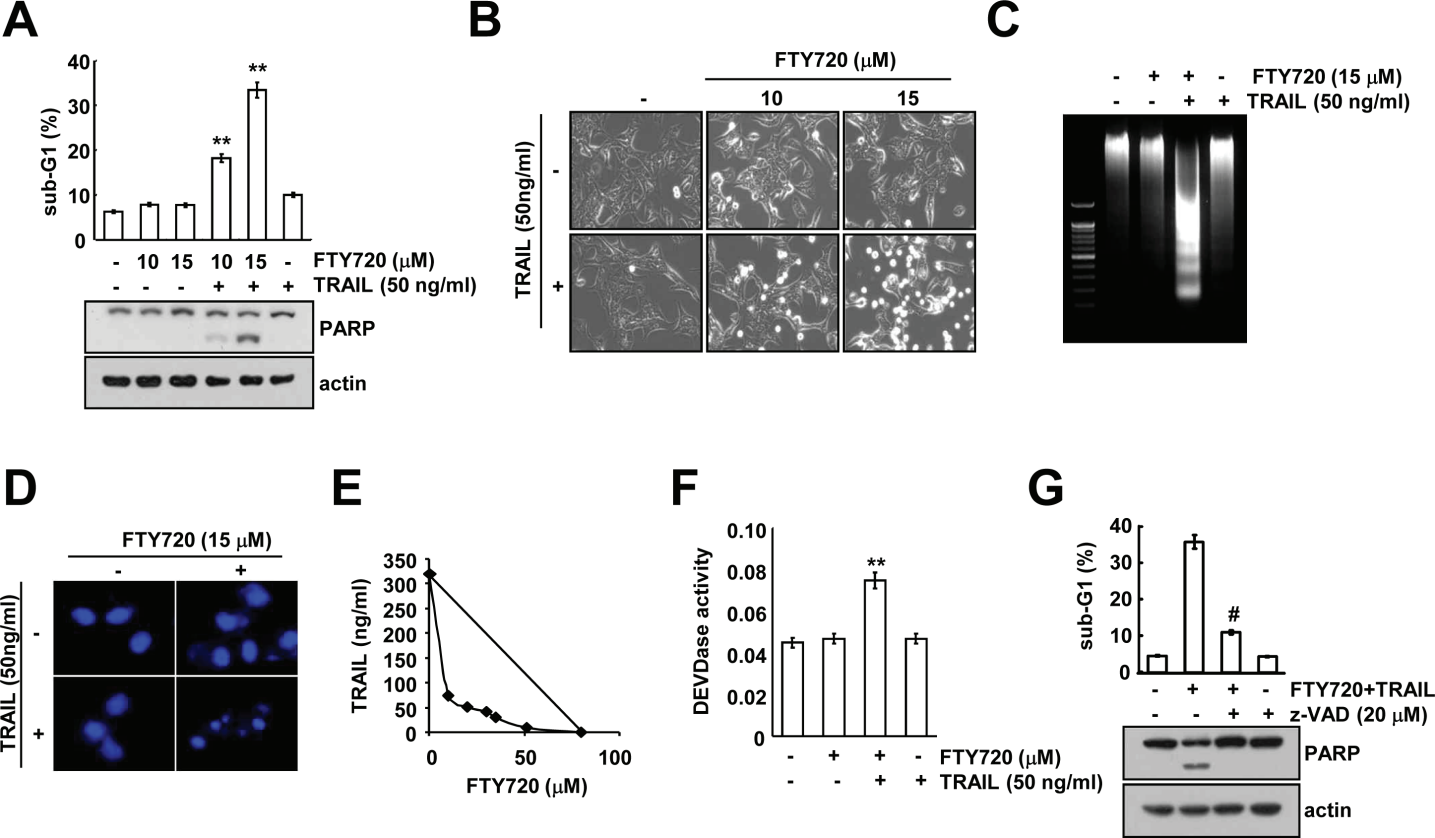
FTY720 sensitizes Caki cells to TRAIL-mediated apoptosis **(A)** Caki cells were treated with 50 ng/ml TRAIL in the presence or absence of the indicated concentrations of FTY720 for 24 h. The sub-G1 fraction was measured by flow cytometry as an indicator of the level of apoptosis. The protein expression levels of PARP were determined by western blotting. The level of actin was used as a loading control. **(B–D)** Caki cells were treated with 50 ng/ml TRAIL in the presence or absence of the indicated concentrations of FTY720 for 24 h. The cell morphology was examined using interference light microscopy (B). Fragmented DNA was extracted and analyzed on a 2% agarose gel (C). The condensation and fragmentation of the nuclei were detected by 4′, 6′-diamidino-2-phenylindole staining (D). **(E)** Isoboles were obtained by plotting the combined concentrations of each drug required to produce 50% cell death. The straight line connecting the IC_50_ values obtained for the two agents when applied alone corresponded to the addition of their independent effects. Values below this line indicate synergy, whereas values above this line indicate antagonism. **(F)** Caki cells were treated with 50 ng/ml TRAIL in the presence or absence of 15 μM FTY720 for 24 h. Caspase activities were determined with colorimetric assays using caspase-3 (DEVDase) assay kits. **(G)** Caki cells were treated with 15 μM FTY720 plus 50 ng/ml TRAIL for 24 h in the presence or absence of 20 μM z-VAD-fmk (z-VAD). The sub-G1 fraction was measured by flow cytometry. The protein expression levels of PARP and actin were determined by western blotting. The level of actin was used as a loading control. The values in A, F, and G represent the mean ± SD from three independent samples. **p < 0.01 compared to the control. ^#^*p* < 0.01 compared to the co-treatment of FTY720 and TRAIL.

FTY720 is known to have anti-cancer effects in several types of cancer cells [9, 10]. Therefore, we investigated whether FTY720 can sensitize human renal carcinoma Caki cells to TRAIL-mediated apoptosis. Neither FTY720 nor TRAIL alone had any effect on apoptosis, but combined treatment with both FTY720 and TRAIL markedly enhanced the sub-G1 population and PARP cleavage, which are markers of apoptosis, in a dose-dependent manner; they also induced morphological changes (Figure 1A and 1B). Next, we examined whether combined treatment with FTY720 and TRAIL induces DNA fragmentation. We analyzed the DNA fragmentation induced by the combined treatment with FTY720 and TRAIL (Figure 1C) and detected the typical apoptotic nuclei (Figure 1D). Therefore, we examined whether combined treatment with FTY720 and TRAIL have synergistic effects. Combined treatment with various concentrations of FTY720 and TRAIL markedly reduced cell viability. The isobologram analysis suggested that the combined treatment with FTY720 and TRAIL has synergistic effects (Figure 1E). In addition, as shown in Figure 1F, FTY720 plus TRAIL increased caspase 2, 3, 8, and 9 activation (Figure 1F, and Supplementary Figure S1), and the pan-caspase inhibitor z-VAD markedly inhibited apoptosis in the FTY720 plus TRAIL-treated cells (Figure 1G). Therefore, these data indicate that combined treatment with FTY720 and TRAIL can induce caspase-dependent apoptosis in human renal carcinoma Caki cells.

### FTY720 plus TRAIL induces apoptosis in other cancer cells, but not in normal cells

**Figure 2:**
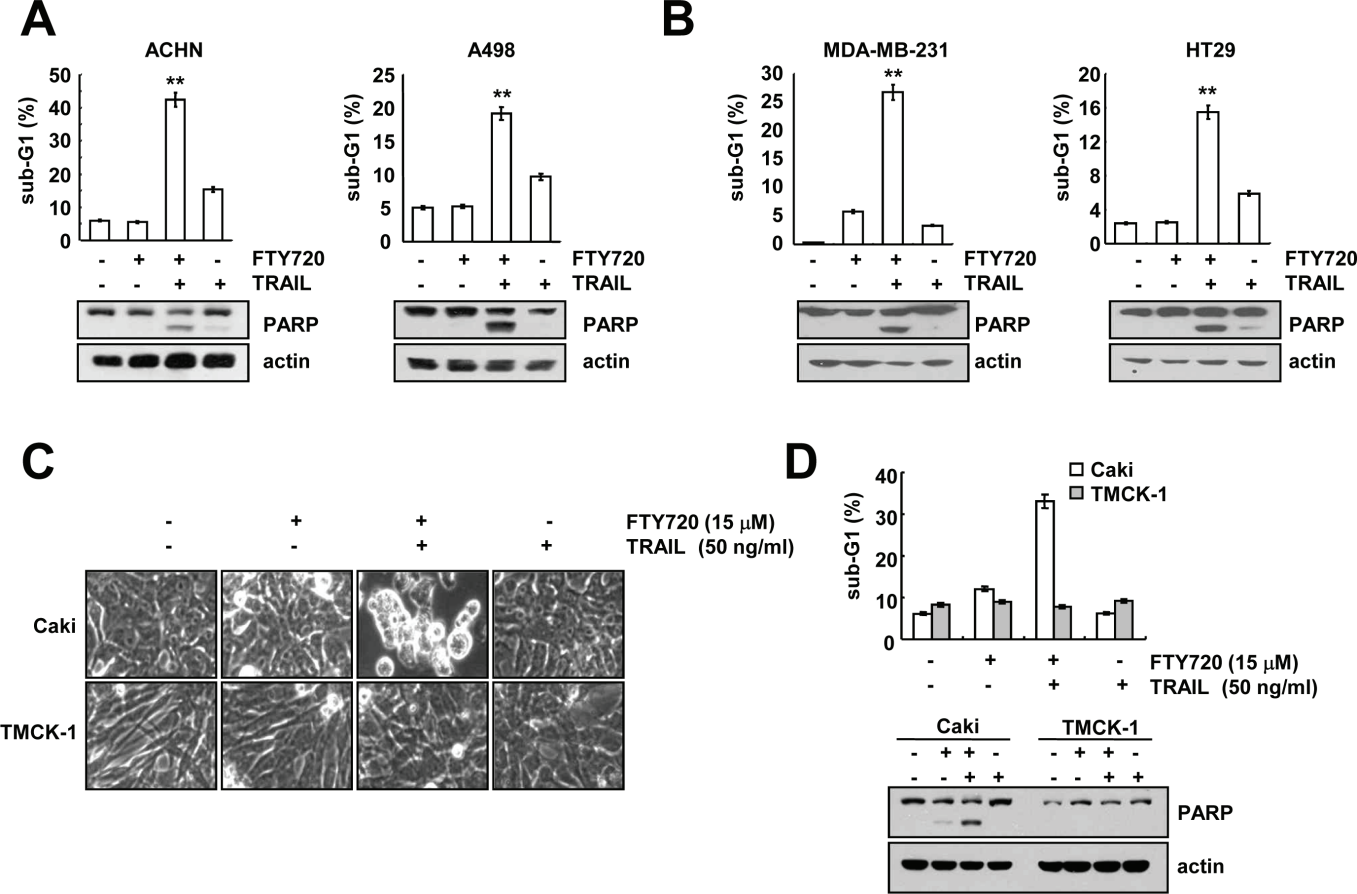
The effects of combined treatment with FTY720 plus TRAIL on apoptosis in other carcinoma cell lines and normal cells **(A and B)** Renal carcinoma (ACHN and A498), breast carcinoma (MDA-MB231), and colon carcinoma (HT29) cells were treated with 50 ng/ml TRAIL in the presence or absence of 15 μM FTY720 for 24 h. The level of apoptosis was assessed by measuring the sub-G1 fraction using flow cytometry. The protein expression levels of PARP and actin were determined by western blotting. The level of actin was used as the loading control. **(C and D)** Caki and TMCK-1 cells were treated with 50 ng/ml TRAIL in the presence or absence of 15 μM FTY720 for 24 h. The cell morphology was examined using interference light microscopy (C). The level of apoptosis was analyzed by measuring the sub-G1 fraction by flow cytometry (D, upper panel). The protein expression levels of PARP and actin were determined by western blotting. The level of actin was used as a loading control (D, lower panel). The values in A, B, and D represent the mean ± SD from three independent samples. **p < 0.01 compared to control.

To investigate the effects of FTY720 on TRAIL-mediated apoptosis, we co-treated other cancer cells with FTY720 and TRAIL. Combined treatment with FTY720 and TRAIL markedly induced apoptosis in renal cancer cells (ACHN and A498), human breast carcinoma cells (MDA-MB-231 cells) and human colon carcinoma (HT29) cells (Figure 2A and 2B). In contrast, combined treatment with FTY720 and TRAIL produced no morphological changes and had no effect on the induction of the sub-G1 population and PARP cleavage in normal mouse kidney cells (TMCK-1) (Figure 2C and 2D). These data indicate that combined treatment with FTY720 and TRAIL might induce apoptosis in cancer cells, but not in normal cells.

### Combined treatment with FTY720 and TRAIL inhibits tumor growth *in vivo*

**Figure 3:**
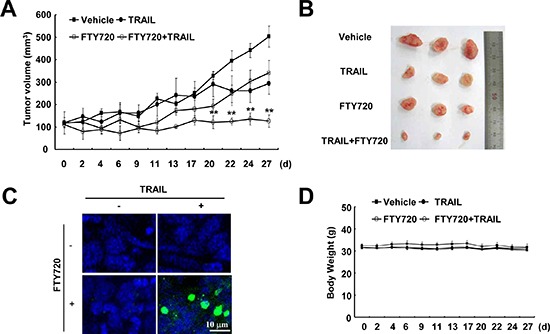
Tumor growth *in vivo* is reduced by the combined treatment with FTY720 and TRAIL Nude mice were subcutaneously inoculated with Caki cells. Tumor volume was monitored during the following treatments: vehicle, FTY720 (7.5 mg/kg; i.p.), GST-TRAIL (3 mg/kg, i.p.), or FTY720 plus TRAIL for 27 days. **(A)** The graph shows changes in the tumor volume. There were 7 animals per group. The data are the means ± SE (*n* = 7). **(B)** The size of the dissected-out tumors are shown. **(C)** Representative images of tumor sections analyzed by the TUNEL assay. Nuclear staining was performed with DAPI. **(D)** Bodyweight changes during the experiment. The values in A and D represent the mean ± SD. **p < 0.01 compared to vehicle.

Next, we investigated whether combined treatment with FTY720 and TRAIL had anti-cancer effects in an *in vivo* xenograft model. Mice bearing tumors were treated with FTY720 alone, TRAIL alone, and FTY720 plus TRAIL. Combined treatment with FTY720 and TRAIL was found to markedly inhibit tumor growth compared with the vehicle control, FTY720 alone, or TRAIL alone (Figure 3A and 3B). Furthermore, we detected cell death using a TUNEL assay in FTY720 and TRAIL-treated samples (Figure 3C). In contrast, FTY720 and TRAIL treatment had no effect on the mouse weight (Figure 3D). These data suggest that combined treatment with FTY720 and TRAIL inhibits tumor growth and induces apoptosis *in vivo*.

### Up-regulation of DR5 is associated with FTY720 and TRAIL-mediated apoptosis

**Figure 4:**
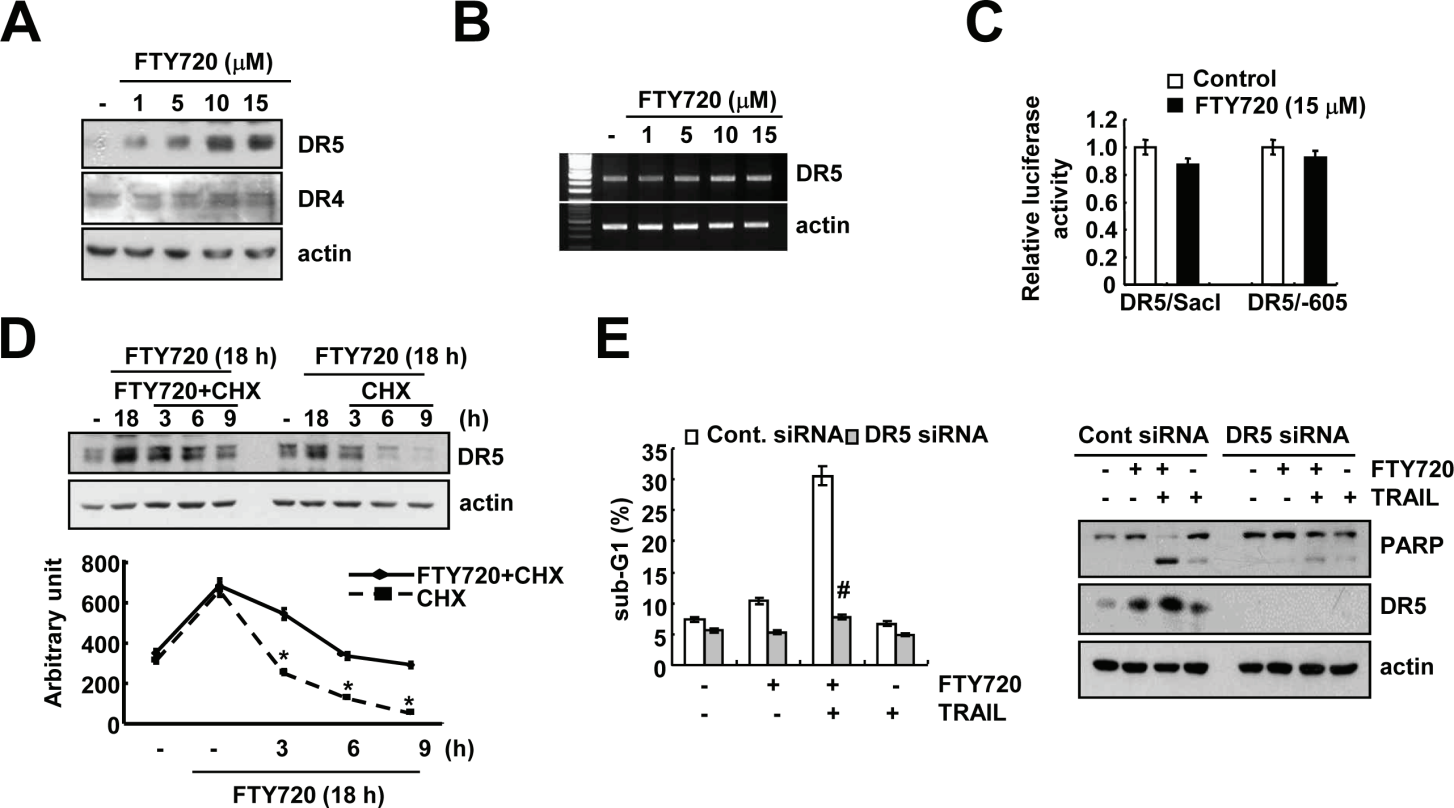
DR5 up-regulation by FTY720 contributes to the sensitization of Caki cells to TRAIL-mediated apoptosis **(A)** Caki cells were treated with the indicated concentrations of FTY720 for 24 h. The protein expression levels of DR5, DR4, and actin were determined by western blotting. The level of actin was used as a loading control. **(B)** Caki cells were treated with the indicated concentrations of FTY720 for 24 h. DR5 mRNA was determined using RT-PCR. **(C)** Caki cells were transiently transfected with plasmids (DR5/SacI and DR5/−605) harboring the luciferase gene under the control of the DR5 promoter. They were then treated with 15 μM FTY720 for 18 h. The cells were lysed, and the luciferase activity was measured. **(D)** Caki cells were treated with 15 μM FTY720 for 18 h, washed with PBS, and then treated with 20 μg/ml cyclohexamide (CHX) in the presence or absence of 15 μM FTY720 for the indicated time periods. DR5 and actin protein levels were determined by western blotting. Actin expression was used as the loading control. The band intensity of the DR5 protein was measured using the public-domain JAVA image-processing program ImageJ (http://rsb.info.nih.gov/ij). **(E)** Caki cells were transiently transfected with DR5 siRNA and then treated with 50 ng/ml TRAIL in the presence or absence of 15 μM FTY720 for 24 h. The sub-G1 fraction was measured by flow cytometry. The protein expression levels of PARP, DR5 and actin were determined by western blotting. The level of actin was used as a loading control. The values in (C, D and E) represent the mean ± SD from three independent samples. *p < 0.05 compared to FTY720 plus CHX. ^#^*p* < 0.01 compared to FTY720 plus TRAIL-treated Cont.siRNA.

Death receptors (DRs) play key roles in TRAIL-mediated apoptosis [22, 24]. Therefore, we determine whether FTY720 modulates the expression of DRs. FTY720 markedly induces DR5 expression, but not DR4 expression (Figure 4A). Next, we investigated whether FTY720 modulates DR5 expression at the transcriptional level. As shown in Figure 4B and 4C, FTY720 did not induce DR5 mRNA expression or promoter activity. Furthermore, FTY720 had no effect on the expression of the C/EBP homologous protein (CHOP), which is an important transcription factor that is involved in the regulation of DR5 mRNA expression (Supplementary Figure S2). Therefore, we investigated whether FTY720 modulates the protein stability of DR5. To investigate this possibility, Caki cells were treated with FTY720 for 18 h, washed with FTY720, and then treated with or without FTY720 in the presence of 20 μg/ml cycloheximide (CHX) for the various indicated times. FTY720 was found to increase DR5 protein stability in Caki cells (Figure 4D). Next, to confirm the significance of the up-regulation of DR5 expression, Caki cells were transiently transfected with DR5 siRNA. The down-regulation of DR5 by siRNA markedly inhibited apoptosis caused by the combined treatment with FTY720 and TRAIL and PARP cleavage (Figure 4E). These results indicate that FTY720 induces the up-regulation of DR5 protein expression at the post-translational level and that the FTY720-mediated DR5 up-regulation is involved in the effects of FTY720 on TRAIL sensitization.

### The down-regulation of Mcl-1 is associated with FTY720 and TRAIL-mediated apoptosis

**Figure 5:**
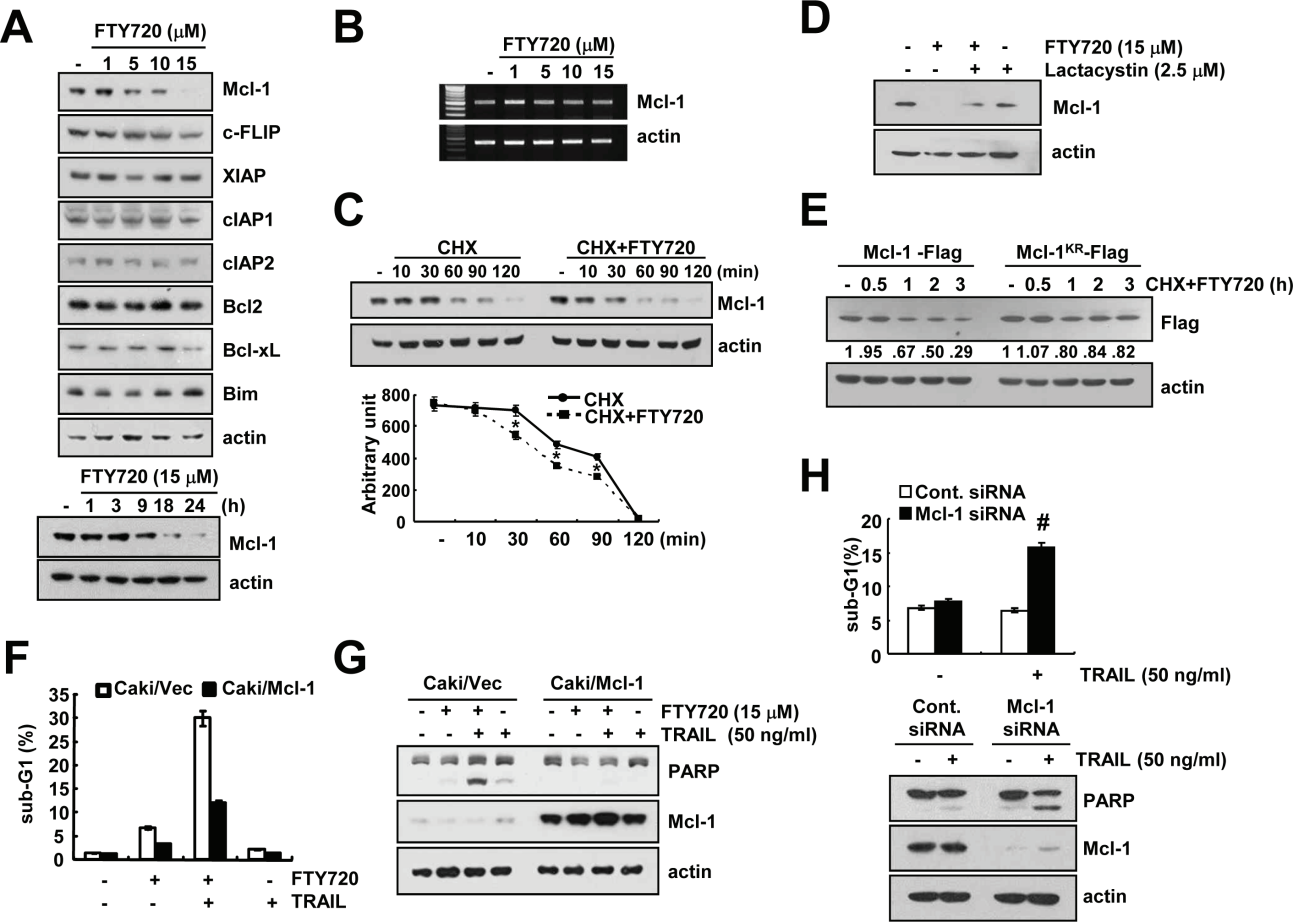
The down-regulation of Mcl-1 by FTY720 is associated with the induction of TRAIL-mediated apoptosis **(A)** Caki cells were treated with the indicated concentrations of FTY720 for 24 h (upper panel) or the indicated time periods (lower panel). The protein expression levels of Mcl-1, c-FLIP, XIAP, cIAP1, cIAP2, Bcl-2, Bcl-xL, Bim, and actin were determined by western blotting. **(B)** Caki cells were treated with the indicated concentrations of FTY720 for 24 h. The mRNA expression levels of Mcl-1 and actin were determined by RT-PCR. **(C)** Caki cells were treated with or without 15 μM FTY720 in the presence of cyclohexamide (CHX) (20 μg/ml) for the indicated time periods. The Mcl-1 and actin protein levels were determined by western blotting. Actin expression was used as a loading control. The band intensity of the Mcl-1 protein was measured using the public domain JAVA image-processing program ImageJ (http://rsb.info.nih.gov/ij). **(D)** Caki cells were pretreated with 2.5 μM lactacystin, and then treated with 15 μM FTY720 for 24 h. The protein expression levels of Mcl-1 and actin were determined by western blotting. Actin expression was used as a loading control. **(E)** Caki cells were transiently transfected with Flag-Mcl-1 and Flag-Mcl-1^KR^. Twenty-four hours after transfection, the cells were treated with 20 μg/ml cyclohexamide (CHX) and 15 μM FTY720 for the indicated time periods. Mcl-1 and actin protein levels were determined by western blotting. Actin expression was used as the loading control. **(F and G)** Vector cells (Caki/vector) and Mcl-1 overexpressed cells (Caki/Mcl-1) were treated with 50 ng/ml TRAIL in the presence or absence of 15 μM FTY720 for 24 h. The level of apoptosis was analyzed by the sub-G1 fraction using flow cytometry (F). The PARP, Mcl-1 and actin protein levels were determined by western blotting. Actin expression was used as a loading control (G). **(H)** Caki cells were transiently transfected with Mcl-1 siRNA and then treated with 50 ng/ml TRAIL for 24 h. The sub-G1 fraction was measured by flow cytometry. The protein expression levels of PARP, Mcl-1 and actin were determined by western blotting. The level of actin was used as a loading control. The values in (C, F and H) represent the mean ± SD from three independent samples. *p < 0.05 compared to CHX. ^#^*p* < 0.05 compared to TRAIL-treated Cont.siRNA.

Next, we investigated whether FTY720 modulates the expression of apoptosis regulatory proteins. The detected apoptosis regulatory proteins did not markedly change their expression levels, but Mcl-1 expression was reduced in a dose-dependent manner in the FTY720-treated cells (Figure 5A). FTY720 induced the down-regulation of Mcl-1 protein expression within 9 h (Figure 5A). Therefore, we examined whether FTY720 modulates Mcl-1 mRNA expression. However, FTY720 had no effect on Mcl-1 mRNA expression (Figure 5B).

When Caki cells were treated with or without FTY720 in the presence of 20 μg/ml CHX for the various indicated time periods, FTY720 decreased the Mcl-1 protein stability in Caki cells (Figure 5C). Previous studies reported that the degradation of Mcl-1 was mainly modulated by the ubiquitin-proteasome pathway [40]. Therefore, we investigated whether FTY720 also modulates Mcl-1 protein expression via the ubiquitin-proteasome pathway. First, we determine the effect of the proteasome inhibitor (lactacystin) on FTY720-induced Mcl-1 degradation. As shown in Figure 5D, lactacystin markedly reversed the FTY720-induced down-regulation of Mcl-1. Next, to determine whether the Mcl-1 degradation caused by FTY720 treatment is dependent on ubiquitination, Caki cells were transiently transfected with Flag-Mcl-1 or Flag-Mcl-1^KR^, in which all 14 lysine residues were replaced with arginine. As shown in Figure 5E, CHX and FTY720 treatment led to the degradation of the Flag-Mcl-1 protein; the degradation of the Flag-Mcl-1^KR^ protein is slower than the degradation of Flag-Mcl-1. These data indicate that FTY720-mediated Mcl-1 degradation is mainly ubiquitin-dependent, but that the involvement of the ubiquitin-independent pathway might also be associated with the degradation of Mcl-1 proteins. To investigate the mechanism of Mcl-1 degradation, we examined whether Mcl-1 expression was dependent on mitogen activated protein kinase (MAPK) activation in the FTY720-treated cells. However, the use of MAPK inhibitors did not block Mcl-1 down-regulation in the FTY720-treated cells (Supplementary Figure S3). Next, we investigated whether the down-regulation of Mcl-1 is critical for apoptosis following combined treatment with FTY720 and TRAIL. When Mcl-1 was over-expressed, the induction of apoptosis and cleavage of PARP caused by combined treatment with FTY720 and TRAIL decreased (Figure 5F and 5G). To confirm the importance of the down-regulation of Mcl-1 expression on TRAIL sensitization, Caki cells were transiently transfected with Mcl-1 siRNA. The down-regulation of Mcl-1 expression by siRNA sensitized TRAIL-mediated apoptosis (Figure 5H). These results indicate that the down-regulation of Mcl-1 has an important role on FTY720-mediated TRAIL sensitization.

### The effect of FTY720-induced ROS production on TRAIL sensitization

**Figure 6:**
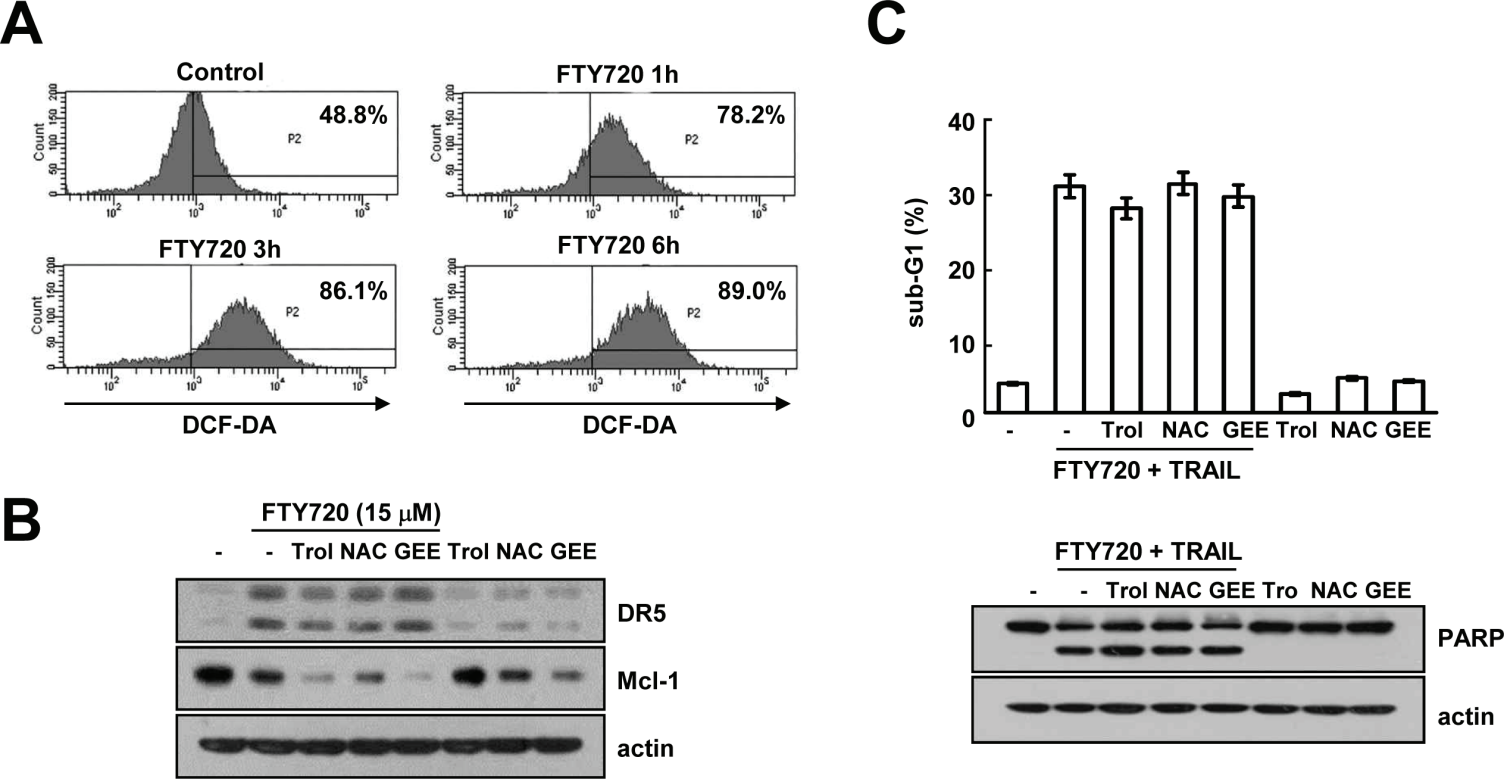
FTY720 and TRAIL-mediated apoptosis is independent of ROS signaling in Caki cells **(A)** Caki cells were treated with 15 μM FTY720 for the indicated time periods and loaded with H_2_DCF-DA fluorescent dye. The H_2_DCF-DA fluorescence intensity was detected by flow cytometry. **(B)** Caki cells were pretreated with 200 μM trolox (Trol), 5 mM NAC, and 2 mM GEE for 30 min and then stimulated with 15 μM FTY720 for 24 h. The protein expression levels of DR5, Mcl-1 and actin were determined by western blotting. The level of actin was used as a loading control. **(C)** Caki cells were pretreated with 200 μM trolox (Trol), 5 mM NAC, and 2 mM GEE for 30 min, and then stimulated with 15 μM FTY720 plus 50 ng/ml TRAIL for 24 h. Apoptosis was analyzed in the sub-G1 population by FACS analysis. The protein expression levels of PARP and actin were determined by western blotting. The level of actin was used as the loading control. The values in (C) represent the mean ± SD from three independent samples.

FTY720 induced intracellular reactive oxygen species (ROS) production in cancer cells [10, 41]. The induction of higher ROS levels is known to play an important role in TRAIL sensitization [42, 43]. Therefore, we investigated whether the induction of ROS levels by FTY720 is associated with TRAIL sensitization. The levels of intracellular ROS production are markedly up-regulated in FTY720-treated cells (Figure 6A). However, ROS scavengers [trolox (trol), N-acetyl-L-cysteine (NAC), and glutathione ethyl ester (GEE)] were found to have no effect on FTY720 induced-DR5 up-regulation and Mcl-1 down-regulation (Figure 6B). Furthermore, ROS scavengers did not affect apoptosis resulting from the combined treatment with FTY720 and TRAIL or the PARP cleavage (Figure 6C). These results suggest that effect of FTY720 on TRAIL sensitization is independent of the level of ROS production.

Taken together, our results demonstrate that FTY720 sensitizes cells to TRAIL-induced apoptosis through the up-regulation of DR5 and down-regulation of Mcl-1 expression in human renal Caki cells.

## DISCUSSION

In this study, we demonstrated, for the first time, that FTY720 enhances TRAIL-mediated apoptosis in cancer cells, but not in normal cells. Furthermore, combined treatment with FTY720 and TRAIL reduced the tumor volume and induced apoptosis in a xenograft model. We found that the mechanism of FTY720-mediated TRAIL sensitization is associated with the up-regulation of DR5 protein stability and down-regulation of Mcl-1 protein stability. Although FTY720 markedly increased the intracellular ROS levels, FTY720-mediated TRAIL sensitization was found to be independent of ROS signaling. These findings suggest that FTY720 could be an attractive drug for TRAIL-sensitization.

FTY720 activates sphingosine-1-phosphate (S1P) receptors following phosphorylation by sphingosine kinase 2. Therefore, we investigated the involvement of S1P receptor signaling by assessing the impact of phospho-FTY720 on TRAIL sensitization. In contrast to FTY720, phospho-FTY720 had no effect on the TRAIL sensitization, up-regulation of DR5 expression, or down-regulation of Mcl-1 expression (Supplementary Figure S4A). Furthermore, sphingosine kinase inhibitors [N, N-dimethylsphingosine (DMS) and sphingosine kinase inhibitor (SKI)-178] and the down-regulation of sphingosine kinase 1 (SK1) by siRNA also had no effect on TRAIL sensitization (Supplementary Figure S4B and Supplementary S4C). These data suggest that the effects of FTY720 on TRAIL sensitization are independent of S1P receptor signaling and the inhibition of sphingosine kinase 1.

FTY720 has been shown to have anti-cancer effects in several cancer cells, and its major anti-cancer effects are independent of S1P receptor signaling. First, FTY720 induces apoptosis in human hepatoma cells through the activation of PKC δ signaling [41]. FTY720 induces ROS production via the down-regulation of anti-oxidant enzyme (GST-π) expression and then activates PKCδ in human hepatoma cells [41]. However, the PKC δ inhibitor (rottlerin) cannot reverse apoptosis in the FTY720 and TRAIL-treated Caki cells (Supplementary Figure S4D). In our previous study, we reported that rottlerin induced apoptosis in human colon carcinoma cells through the up-regulation of DR5 and NAG-1 expression in a PKC δ–independent manner [44, 45]. Therefore, to confirm the effect of PKC δ, Caki cells were transiently transfected with PKC δ siRNA and then treated with FTY720 plus TRAIL. The down-regulation of PKC δ by siRNA did not rescue apoptosis in FTY720 plus TRAIL-treated cells (data not shown). Therefore, the anti-cancer effects of FTY720 are independent of the activation of PKC δ in human renal carcinoma Caki cells. Second, FTY720 activates protein phosphatase (PP)2A. FTY720 induces cell death in chronic lymphocytic leukemia B cells and leukemia T cells via the activation of PP2A [17, 46]. However, FTY720 also induces caspase-independent cell death in acute lymphoblastic leukemia cells, but the effects of FTY720 are independent of PP2A activation. In other words, the mechanism of FTY720 could differ in different cell types. In our study, we also investigated whether PP2A activity is involved in FTY720 and TRAIL-mediated apoptosis. As shown in Supplementary Figure. S4D, a PP2A inhibitor (okadaic acid) had no effect on apoptosis. In addition, FTY720 also induced intracellular calcium concentrations through phospholipase C activation, which induced apoptosis in human promyelocytic leukemia cells [18]. We found that the FTY720-induced TRAIL sensitization is independent of phospholipase C activation (Supplementary Figure S4D). Although we failed to identify the intracellular mechanism of FTY720, FTY720 could sensitize human renal carcinoma (Caki, ACHN, and A498 cells), human breast carcinoma (MDA-MB-231), and human colon carcinoma (HT29) cells to TRAIL-mediated apoptosis. Therefore, this novel mechanism might be related to the anti-cancer effects of FTY720 in cancer cells. However, the mechanism by which FTY720 sensitizes cells to TRAIL-mediated apoptosis remains unclear and will require further investigation.

FTY720 induced the up-regulation of DR5 protein expression at the post-translational level (Figure 4D). Recently, Casitas B-lineage lymphoma (Cbl) was found to modulate the degradation of DR proteins. Cbl has been known to be a multi-adaptor protein and E3 ligase. Thus, receptor tyrosine kinases are ubiquitinated by Cbl and then degraded by proteasome or lysosome [47, 48]. Song *et al*. reported that c-Cbl is responsible for the degradation of DRs by proteasomes and lysosomes in prostate carcinoma cells [49]. Yan *et al*., also reported that the down-regulation of Cbl-b by bufalin induced the up-regulation of DR expression in breast carcinoma cells [50]. In our study, we also detected a down-regulation of c-Cbl in FTY720-treated cells (data not shown). However, this down-regulation of c-Cbl had no effect on DR5 expression or TRAIL sensitization in FTY720-treated cells (data not shown). Therefore, these results indicate that the up-regulation of DR5 by FTY720 is independent of the down-regulation of c-Cbl expression.

Collectively, these results suggest that FTY720 sensitizes TRAIL-mediated apoptosis through the up-regulation of DR5 expression and down-regulation of Mcl-1 expression in the human renal Caki cell line. Therefore, we suggest that FTY720 may be effectively used as a sensitizer of TRAIL.

## MATERIALS AND METHODS

### Cell culture and materials

Human renal carcinoma (Caki, ACHN, and A498), human breast carcinoma cells (MDA-MB-231), and human colon carcinoma cells (HT29) were obtained from the American Type Culture Collection (Manassas, VA, USA). The mouse kidney cells (TMCK-1) was a gift from Dr. T.J. Lee (Yeungnam University, Korea). The culture medium used throughout these experiments was Dulbecco's modified Eagle's medium (DMEM) or RPMI containing 10% fetal bovine serum (FBS), 20 mM HEPES buffer and 100 μg/mL gentamycin. FTY720 and phospho-FTY720 were purchased from Echelon Biosciences (Salt Lake City, UT, USA). The recombinant human TRAIL was purchased from KOMA Biotech (Seoul, Korea), and z-VAD-fmk, sphingosine kinase inhibitor (SKI), N-acetyl-L-cysteine (NAC) and Trolox was obtained from Calbiochem (San Diego, CA, USA). Cyclohexamide, lactacystin, and glutathione ethyl ester (GEE) were purchased from Sigma Chemical Co. (St. Louis, MO, USA). N, N-dimethylsphingosine (DMS) was purchased from Cayman Chemical Company (Ann Arbor, MI, USA). GST-TRAIL cDNA plasmid was a gift from Dr. Kim YS (Ajou university, Korea). Anti-DR5, anti-DR4 anti-Bcl-2, anti-Bcl-xL, anti-Mcl-1, anti-cIAP1, anti-cIAP2, anti-XIAP, and anti-PARP antibodies were purchased from Santa Cruz Biotechnology (Santa Cruz, CA, USA). Anti-c-FLIP antibody was obtained from ALEXIS Corporation (San Diego, CA, USA). Anti-Bim antibody was purchased from Millipore Corporation (Billerica, MA, USA). Anti-actin and anti-Flag antibodies were obtained from Sigma (St. Louis, MO, USA). Flag-Mcl-1 (plasmid number: 32978) and Flag-Mcl-1^KR^ (plasmid number: 32979), which was deposited by Stewart, was purchased from addgene (Cambridge, MA, USA) [51].

### Flow cytometry analysis

For flow cytometry, the cells were resuspended in 100 μl of phosphate-buffered saline (PBS), and 200 μl of 95% ethanol was added while the cells were being vortexed. Then, the cells were incubated at 4°C for 1 h, washed with PBS, resuspended in 250 μl of 1.12% sodium citrate buffer (pH 8.4) with 12.5 μg of RNase and incubated for an additional 30 min at 37°C. The cellular DNA was then stained by adding 250 μl of a propidium iodide solution (50 μg/ml) to the cells for 30 min at room temperature. The stained cells were analyzed by fluorescent-activated cell sorting on a FACScan flow cytometer to determine the relative DNA content, which was based on the red fluorescence intensity.

### Western blot analysis

For the Western blotting experiments, the cells were washed with cold PBS and lysed on ice in modified RIPA buffer (50 mM Tris-HCl pH 7.4, 1% NP-40, 0.25% Na-deoxycholate, 150 mM NaCl, 1 mM Na_3_VO_4_, and 1 mM NaF) containing protease inhibitors (100 μM phenylmethylsulfonyl fluoride, 10 μg/ml leupeptin, 10 μg/ml pepstatin, and 2 mM EDTA). The lysates were centrifuged at 10,000 × *g* for 10 min at 4°C, and the supernatant fractions were collected. The proteins were separated by SDS-PAGE electrophoresis and transferred to Immobilon-P membranes. The specific proteins were detected using an enhanced chemiluminescence (ECL) Western blotting kit according to the manufacturer's instructions.

### DNA fragmentation assay

After treatment with FTY720 plus TRAIL, Caki cells were lysed in a buffer containing 10 mM Tris (pH 7.4), 150 mM NaCl, 5 mM EDTA, and 0.5% Triton X-100 for 30 min on ice. Lysates were vortexed and cleared by centrifugation at 10,000 × g for 20 min. Fragmented DNA in the supernatant was extracted with an equal volume of neutral phenol:chloroform:isoamyl alcohol mixture (25:24:1) and analyzed electrophoretically on 2% agarose gels containing 0.1 μg/ml of ethidium bromide.

### 4′, 6′-diamidino-2-phenylindole staining (DAPI) for nuclei condensation and fragmentation

To examine cellular nuclei, the cells were fixed with 1% paraformaldehyde on glass slides for 30 min at room temperature. After the fixation, the cells were washed with PBS and a 300 nM 4′, 6′-diamidino-2-phenylindole solution (Roche, Mannheim, Germany) was added to the fixed cells for 5 min. After the nuclei were stained, the cells were examined by fluorescence microscopy.

### Determination of synergy

The possible synergistic effect of FTY720 and TRAIL was evaluated using the isobologram method. In brief, the cells were treated with different concentrations of FTY720 and TRAIL alone or in combination. After 24 h, XTT assay was employed to measure the cell viability using WelCount Cell Viability Assay Kit (WelGENE, Daegu, Korea). In brief, reagent was added to each well and was then measured with a multi-well plate reader (at 450 nm/690 nm). Relative survival was assessed and the concentration effect curves were used to determine the IC_50_ (the half-maximal inhibitory concentration) values for each drug alone and in combination with a fixed concentration of the second agent [52].

### Asp-Glu-Val-Asp-ase (DEVDase) activity assay

To evaluate DEVDase activity, cell lysates were prepared after their respective treatments with TRAIL in the presence or absence of FTY720. Assays were performed in 96-well microtiter plates by incubating 20 μg of cell lysates in 100 μl of reaction buffer (1% NP-40, 20 mM Tris-HCl, pH 7.5, 137 mM NaCl, 10% glycerol) containing a caspase substrate [Asp-Glu-Val-Asp-chromophore-p-nitroanilide (DVAD-pNA)] at 5 μM. Lysates were incubated at 37°C for 2 h. Thereafter, the absorbance at 405 nm was measured with a spectrophotometer.

### Animal

Male BALB/c-nude mice, aged 5 weeks, were purchased from the Central Lab Animal Inc. (Seoul, Korea). All the mice were allowed 1 week to acclimatize to the surroundings before the experiments, and were kept at 25 ± 2°C, with a relative humidity of 55 ± 5% and a 12 h light–dark cycle. The study protocol was approved by the IRB Keimyung University Ethics Committee.

### *in vivo* xenograft model

Each mouse was subcutaneously (s.c.) injected on each flank with Caki cells (2 × 10^6^). After tumors had grown after approximately 2 weeks, 28 mice were randomly divided into four treatment groups: (1) vehicle alone, (2) FTY720 alone, (3) GST-TRAIL alone, and (4) FTY720 plus GST-TRAIL. FTY720 and GST-TRAIL were administered at 7.5 mg/kg and 3 mg/kg, respectively. FTY720 and GST-TRIAL were prepared in PBS (pH 7.4). The mice received an intraperitoneal (i.p.) injection of vehicle, FTY720 and GST-TRAIL. Treatment was administered three times a week for 4 weeks. The tumor size was measured three times a week using a Vernier's caliper (Mytutoyo Co., Japan) to measure two perpendicular diameters, and the tumor size was calculated using the equation (length × width^2^)/2. The animals were sacrificed by cervical dislocation, and the tumors were collected for histological analysis. The tumors were fixed in 30% formalin, embedded in OCT compound (Miles Inc., Elkhart, IN, USA) and cut into 20-μm sections using a cryostat (SLEE International, Inc., New York, NY, USA).

### TUNEL assay

Apoptosis in tumor cells was detected by terminal deoxynucleotide transferase (TdT)-mediated dUTP nick-end labeling (TUNEL) assay. It was performed using the ApopTag Fluorescein *In Situ* Apoptosis Detection Kit (Millipore, Billerica, MA, USA) as the manufacturer's protocol.

### Reverse transcription polymerase chain reaction (RT-PCR)

Total RNA was isolated using the TriZol reagent (Life Technologies; Gaithersburg, MD, USA), and the cDNA was prepared using M-MLV reverse transcriptase (Gibco-BRL; Gaithersburg, MD, USA) according to the manufacturers' instructions. The following primers were used for the amplification of human DR5, Mcl-1, and actin: DR5 (sense) 5′-AAG ACC CTT GTG CTC GTT GT-3′ and (antisense) 5′-GAC ACA TTC GAT GTC ACT CCA-3′, Mcl-1 (sense) 5′-GCG ACT GGC AAA GCT TGG CCT CAA-3′ and (antisense) 5′-GTT ACA GCT TGG ATC CCA ACT GCA-3′, and actin (sense) 5′-GGC ATC GTC ACC AAC TGG GAC-3′ and (anti-sense) 5′-CGA TTT CCC GCT CGG CCG TGG-3′. The PCR amplification was carried out using the following cycling conditions: 94°C for 3 min followed by 17 (actin) or 23 cycles (DR5 and Mcl-1) of 94°C for 45 s, 58°C for 45 s, 72°C for 1 min, and a final extension at 72°C for 10 min. The amplified products were separated by electrophoresis on a 1.5% agarose gel and detected under UV light.

### Plasmids, transfection and luciferase assay

The pDR5/SacI plasmid [containing DR5 promoter sequence (−2500/+3)] and pDR5/−605 [containing DR5 promoter sequence (−605/+3)] were a gift from Dr Sakai T (Kyoto Prefectural University). Transient transfection was performed in 6-well plates.

One day before the transfection, Caki cells were plated at approximately 60–80% confluence. The DR5/SacI and DR5/−605 promoter plasmid was transfected into the cells using Lipofectamine™ 2000 (Invitrogen; Carlsbad, CA, USA). To assess the promoter-driven expression of the luciferase gene, the cells were collected and disrupted by sonication in lysis buffer (25 mM Tris-phosphate pH 7.8, 2 mM EDTA, 1% Triton X-100, and 10% glycerol), and aliquots of the supernatants were used to analyze the luciferase activity according to the manufacturer's instructions (Promega; Madison, WI, USA).

### Small interfering RNA (siRNA)

The DR5 siRNA used in this study was purchased from Invitrogen (Calsbad, CA, USA). Mcl-1 siRNA and sphingosine kinase 1 siRNA were purchased from Santa Cruz Biotechnology (Santa Cruz, CA, USA). The siRNA had the following sequences: DR5, AUC AGC AUC GUG UAC AAG GUG UCC C; and green fluorescent protein [GFP (control)], AAG ACC CGC GCC GAG GUG AAG. Cells were transfected with siRNA oligonucleotides using Oligofectamine reagent (Invitrogen, Carlsbad, CA, USA) according to the manufacturer's recommendations.

### Stable transfection in Caki cells

The Caki cells were transfected in a stable manner with the pFLAG-CMV4-Mcl-1, or control plasmid pcDNA 3.1 vector using Lipofectamine2000 as prescribed by the manufacturer (Invitrogen, Carlsbad, CA, USA). After 48 h of incubation, transfected cells were selected in primary cell culture medium containing 700 μg/mL G418 (Invitrogen). After 2 or 3 weeks, single independent clones were randomly isolated, and each individual clone was plated separately. After clonal expansion, cells from each independent clone were tested for expression levels of Mcl-1 by immunoblotting.

### Measurement of reactive oxygen species (ROS)

Intracellular accumulation of ROS was determined using the fluorescent probes 2′, 7′-dichlorodihydrofluorescein diacetate (H_2_DCFDA). H_2_DCFDA is commonly used to measure ROS generation. Caki cells were treated with FTY720, and then cells were stained with the fluorescent dye H_2_DCFDA for an additional 10 min. Then, cells were trypsinized and resuspended in PBS, and fluorescence was measured at specific time intervals with a flow cytometer (Becton–Dickinson; Franklin Lakes, NJ, USA).

### Densitometry

The band intensities were scanned and quantified using the gel analysis plugin for the open source software ImageJ 1.46 (Imaging Processing and Analysis in Java; http://rsb.info.nih.gov/ij).

### Statistical analysis

The data were analyzed using a one-way ANOVA and post-hoc comparisons (Student-Newman-Keuls) using the Statistical Package for Social Sciences 22.0 software (SPSS Inc.; Chicago, IL, USA).

## SUPPLEMENTARY FIGURES


