# Supplementary material for: FTY720 enhances TRAIL-mediated apoptosis by up-regulating DR5 and down-regulating Mcl-1 in cancer cells
Source: Oncotarget. 2015 Mar 23;6(13):11614–26. doi: 10.18632/oncotarget.3426 (PMC4484480; doi:10.18632/oncotarget.3426)
Supplement: Supplementary file 1 [file oncotarget-06-11614-s001.pdf]

SUPPLEMENTARY FIGURES

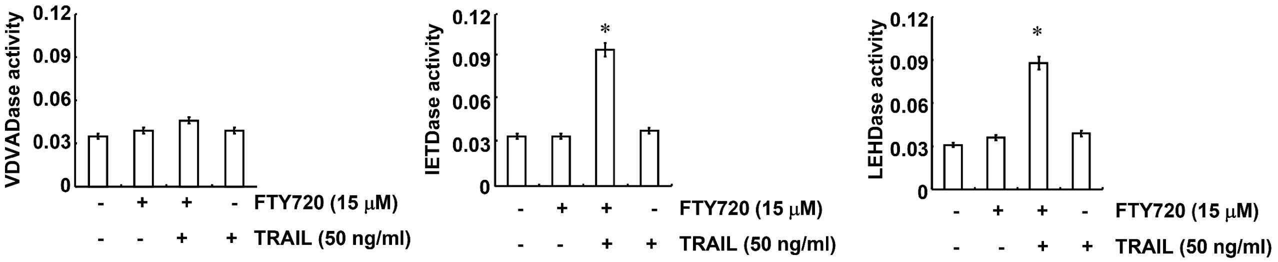

Supplementary Figure S1: The effects of combined treatment with FTY720 and TRAIL on caspase activation. Caki cells were treated with 50 ng/ml TRAIL in the presence or absence of 15 μM FTY720 for 24 h. The resulting caspase activities were determined with colorimetric assays using caspase-2 (VDVADase) assay kits, caspase-8 (IETDase) assay kits and caspase-9 (LEHDase) assay kits. The values represent the mean ± SD from three independent samples. *$p < 0.05$ compared to FTY720 treatment alone.

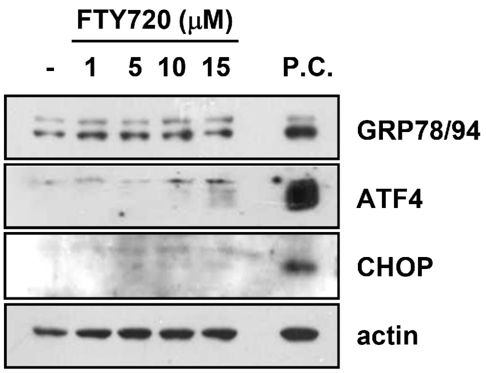

Supplementary Figure S2: FTY720 has no effect on the expression of ER stress-related proteins. Caki cells were treated with the indicated concentrations of FTY720 for 9 h. The protein expression levels of Grp78/94, ATF4, CHOP and actin were determined by western blotting. Actin expression was used as a loading control. Brefeldin A (2 μM) was used as a positive control (P.C.).

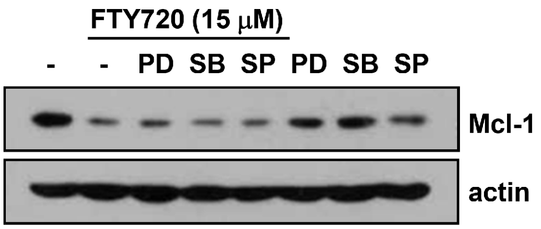

Supplementary Figure S3: The effects of MAPKs inhibitors on the FTY720-induced down-regulation of Mcl-1 expression. Caki cells were pretreated with inhibitors of MAPKs at different concentrations [50 μM MEK1/2 inhibitor PD98059 (PD); 10 μM p38 MAP kinase inhibitor SB203580 (SB); or 10 μM JNK inhibitor SP600125 (SP)], and then treated with 15 μM FTY720 for 24 h. The protein expression levels of Mcl-1 and actin were determined by western blotting. Actin expression was used as a loading control.

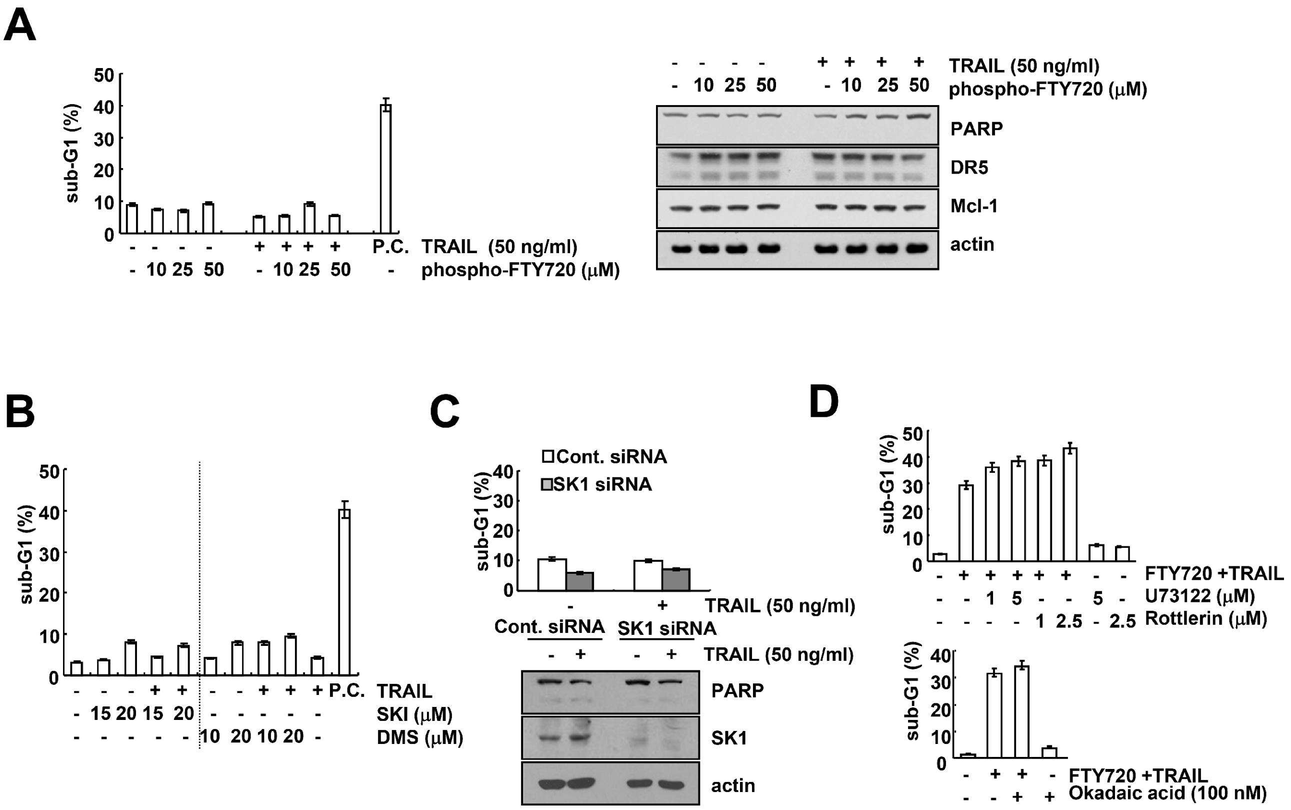

Supplementary Figure S4: Effects of pharmacologic inhibitors of various signaling molecules on FTY720 and TRAIL-mediated apoptosis. **(A)** Caki cells were treated with 50 ng/ml TRAIL in the presence or absence of the indicated concentrations of phospho-FTY720 for 24 h. **(B)** Caki cells were treated with 50 ng/ml TRAIL in the presence or absence of the indicated concentrations of sphingosine kinase inhibitor (SKI) or N, N, -dimethylsphingosine (DMS). **(C)** Caki cells were transiently transfected with sphingosine kinase 1 (SK1) siRNA and then treated with 50 ng/ml TRAIL for 24 h. **(D)** Caki cells were pretreated with the indicated concentrations of okadaic acid (inhibitor of PP2A), rottlerin (inhibitor of PKC δ), and U73122 (inhibitor of phospholipase C) for 30 min, and then treated with 15 μM FTY720 plus 50 ng/ml TRAIL for 24 h. The sub-G1 fraction was measured by flow cytometry as an indicator of the level of apoptosis (A, B, C and D). The values in A, B, C and D represent the mean ± SD from three independent samples. The protein expression levels of PARP, DR5, Mcl-1, SK1 and actin were determined by western blotting. P.C.; positive control (combined treatment with 15 μM FTY720 plus 50 ng/ml TRAIL).
